# „Ab morgen bitte online“: Vergleich digitaler Rahmenbedingungen der curricularen Lehre an nationalen Universitäts-HNO-Kliniken in Zeiten von COVID-19: Digitale Lehre an nationalen Universitäts-HNO-Kliniken

**DOI:** 10.1007/s00106-020-00939-5

**Published:** 2020-09-14

**Authors:** C. Offergeld, M. Ketterer, M. Neudert, F. Hassepaß, N. Weerda, B. Richter, L. Traser, C. Becker, N. Deeg, A. Knopf, T. Wesarg, A-K. Rauch, T. Jakob, F. Ferver, F. Lang, V. Vielsmeier, S. Hackenberg, M. Diensthuber, M. Praetorius, B. Hofauer, N. Mansour, S. Kuhn, T. Hildenbrand

**Affiliations:** 1 Univ.-HNO-Klinik, Med. Fakultät, Albert-Ludwigs-Universität, Killianstraße 5, 79106 Freiburg, Deutschland; 2 Univ.-HNO-Klinik, Med. Fakultät, Technische Universität Dresden, Dresden, Deutschland; 3 Institut für Musikermedizin, Med. Fakultät, Albert-Ludwigs-Universität, Freiburg, Deutschland; 4 Univ.-HNO-Klinik, Med. Fakultät, Universität Regensburg, Regensburg, Deutschland; 5 Univ.-HNO-Klinik, Med. Fakultät, Julius-Maximilians-Universität, Würzburg, Deutschland; 6 Univ.-HNO-Klinik, Med. Fakultät, , Goethe Universität, Frankfurt/M, Deutschland; 7 Univ.-HNO-Klinik, Med. Fakultät, Ruprecht-Karls-Universität, Heidelberg, Deutschland; 8 Zentrum für Orthopädie und Unfallchirurgie, Universitätsmedizin, Johannes Gutenberg-Universität, Mainz, Deutschland

**Keywords:** Medizinische Lehre, Digitalisierung, HNO-Heilkunde, Covid-19, Rahmenbedingungen Lehre, Medical education, Digitization, Otorhinolaryngology, Covid-19, Educational frame conditions

## Abstract

**Hintergrund:**

Die Corona-Krise beeinflusst nicht nur das professionelle Handeln, sondern auch die Lehre an den Universitäten. Schlagworte wie „E-Learning“ und „Digitalisierung“ suggerieren die Möglichkeit innovativer, ad hoc verfügbarer Lösungsansätze für die Lehre in der aktuellen COVID-19-Situation. Die aktuelle Umstellung auf digitale Lehre ist aber nicht primär durch eine didaktische Sinnhaftigkeit oder institutionelle Strategie, sondern durch äußere Notwendigkeit geprägt.

**Ziel der Arbeit:**

Ziel der Arbeit war die Erfassung der Lehrsituation an nationalen Universitäts-HNO-Kliniken und akademischen Lehrkrankenhäusern zu Beginn des virtuellen Corona-Sommersemesters 2020.

**Material und Methode:**

Ein eigens erstellter Fragebogen zur jeweiligen lokalen Situation, den örtlichen Rahmenbedingungen sowie zu bundesweiten Szenarien wurde an alle 39 nationalen Universitäts-HNO-Kliniken und 20 akademischen Lehrkrankenhäuser mit HNO-Hauptabteilung versandt.

**Ergebnisse:**

Die ausgefüllten Fragebögen von 31 Universitätskliniken (UK) und 10 akademische Lehrkrankenhäuser (ALK) gingen in die Auswertung ein. Es zeigten sich offensichtliche Diskrepanzen zwischen verfügbaren Ressourcen und tatsächlich verfügbaren digitalisierten Lehrinhalten. Weitere Kritikpunkte offenbarten sich in Bezug auf die Kommunikation mit der Medizinischen Fakultät, die digitale Infrastruktur und insbesondere in der oftmals mangelnden Kollaboration mit den zentralen Supportstrukturen, wie Medien‑, Didaktik- und Rechenzentren.

**Schlussfolgerung:**

Es gibt durchaus positive Beispiele für eine gelungene Überführung der Präsenzlehre in das ausschließlich virtuelle Sommersemester 2020 innerhalb der Universitäts-HNO-Kliniken. Mehrheitlich aber überwiegen kritische Einschätzungen der Lehrbeauftragten bzw. Ärztlichen Direktoren gegenüber der aktuellen Lehrsituation. Eine zeitkritische strategische Weiterentwicklung ist dringend erforderlich.

## Abstract (en)

**Background:**

The corona crisis not only affects professional activities but also teaching and learning at universities. Buzzwords, such as e‑learning and digitalization suggest the possibility of innovative teaching approaches that are readily available to solve the problems of teaching in the current COVID-19 pandemic. The current conversion to digital teaching is not primarily driven by didactic rationale or institutional strategy but by external circumstances.

**Objective:**

The aim of the study was to determine the teaching situation at national university ENT clinics and academic teaching hospitals at the start of the virtual corona summer semester in 2020.

**Material and methods:**

A specifically self-designed questionnaire regarding the local situation and conditions as well as nationwide scenarios was sent to all 39 national university ENT clinics and 20 ENT departments at academic teaching hospitals.

**Results:**

A total of 31 university hospitals and 10 academic teaching hospitals took part in the survey. There were obvious discrepancies between available resources and effectively available digital teaching and learning contents. Further criticism was expressed regarding the communication with the medical faculty, the digital infrastructure and particularly the frequent lack of collaboration with central support facilities, such as media, didactics and datacenters.

**Conclusion:**

There are positive examples of successful transformation of classroom teaching to an exclusively virtual summer semester 2020 within the university ENT clinics; however, critical ratings of assistant professors and medical directors regarding the current teaching situation predominated. A time-critical strategic advancement is urgently needed.

„Hochschulen sehen sich digital gut gerüstet für den Start ins Sommersemester“ [20]. Diese Meldung des Stifterverbands vom 20.04.2020 ist das Ergebnis einer Umfrage bei den Hochschulleitungen bezüglich des „Corona-Semesters“ [20]. Interessant ist diese Aussage nicht nur wegen ihres Inhalts, sondern insbesondere wegen des Veröffentlichungsdatums. Am 19.04.2020 war der initiale Übergangszeitraum verstrichen, welcher in eine normale Präsenzlehre zurückführen sollte. An einigen Standorten wurde mit einem relativ kurzen Vorlauf von 7–14 Tagen vonseiten der Universitätsleitungen und der Medizinischen Fakultäten (MF) darauf hingewiesen, dass sämtliche Lehrveranstaltungen im Zeitraum offizieller Semesterbeginn (06.04. bzw.14.04.2020) bis zum 19.04.2020 ausschließlich online durchgeführt werden sollten. Diese Umstellung war nur möglich, wenn auf bereits bestehende digitalisierte Lehrinhalte der Kliniken gebaut werden konnte oder diese mit großem Zeitaufwand kurzfristig digitalisiert wurden. Dies setzt ein existierendes und funktionierendes (interprofessionelles) Teamwork voraus. Da diese Idealvoraussetzungen nicht überall gegeben sind, wurde mancherorts improvisiert. Die Folge war weniger die Entstehung eines wirklichen Online-Angebots, sondern eher einer „notfallbedingten Fernlehre“, bei welcher zeitbedingt zwar Inhalte präsentiert werden, denen aber ein zugrunde liegendes didaktisches Lehrkonzept fehlt. Vorausgesetzt, dass wie im Rahmen der aktuellen COVID-19-Pandemie Vorlesungen, Seminare und Kurse nicht mehr stattfinden können respektive dürfen, sind an Stelle der Präsenzlehre innovative Lehrmethoden angezeigt und mitunter alternativlos [13, 19, 23]. In derartig unerwarteten Situationen sind Institutionen im Vorteil, welche bereits in der Vergangenheit in adäquate Rahmenbedingungen investiert haben.

Als Sonderfall in der medizinischen Lehre gilt, dass gewisse Lehrinhalte und Fähigkeiten ohne die Möglichkeit praktischer (HNO-)Tätigkeiten nur schwer zu vermitteln sind [17]. Diese lassen sich auch durch Standardkonzepte der Universitäten, die überwiegend Vorlesungs- oder Seminarcharakter haben, nur bedingt umsetzen. Allein deshalb muss eine spezifische digitale Infrastruktur bzw. Alternativlösung an einer Universitätsklinik etabliert werden [8, 13, 19, 23].

Seit dem Beginn der Corona-Pandemie war in den Medien immer wieder zu hören, dass Deutschland (hier: die Universitäten) in jeder Hinsicht gut auf eine solche Krise vorbereitet sei(en). Diese Feststellungen stehen allerdings nicht immer im Einklang mit den subjektiven Beobachtungen an medizinischen Lehrbetrieben. Die COVID-19-Pandemie macht deutlich, dass die Bereitstellung digitaler Lehrveranstaltungen innerhalb der jeweiligen MF in sehr unterschiedlichem Maße erfolgt [8, 10, 11, 16]. Mit dem Ziel, einen objektivierbaren Überblick über den Status quo zum Semesterstart zu erlangen, führten wir eine Umfrage an allen 39 nationalen Universitätskliniken (UK) und exemplarisch 20 akademischen Lehrkrankenhäusern (ALK) mit HNO-Hauptabteilung durch.

## Material und Methode

Alle nationalen UK (*n* = 39) und 20 ALK mit HNO-Hauptabteilung (ALK) wurden mit der Bitte um Teilnahme kontaktiert. Die Auswahl der ALK geschah zufällig unter Berücksichtigung einer ausgewogenen geografischen Verteilung. Zusätzlich wurde ein Bundeswehrkrankenhaus angeschrieben, welches in der Auswertung den ALK zugeordnet wurde.

**Figure 1:**
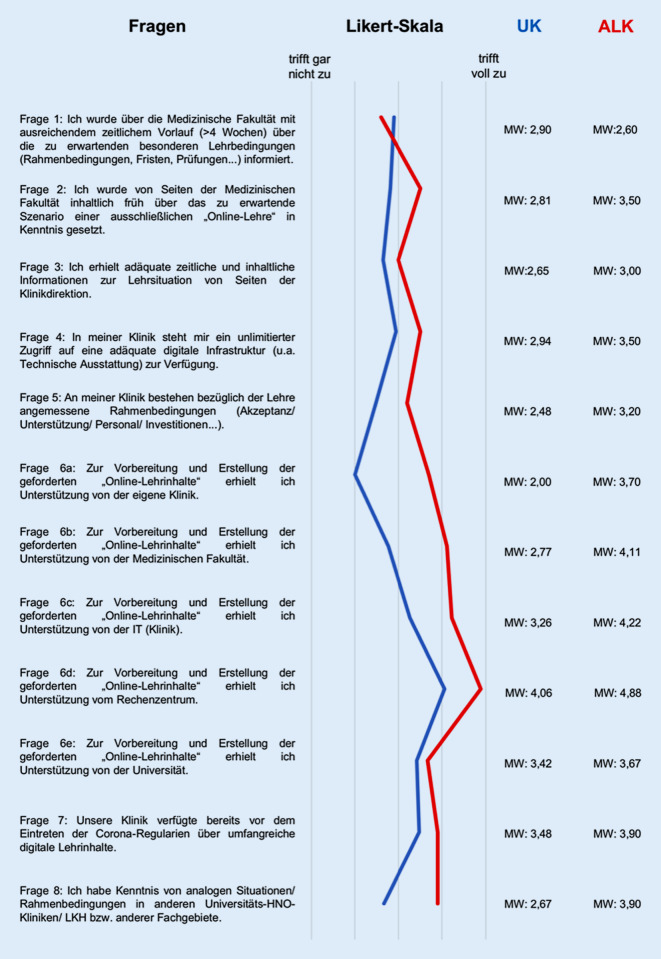

Die Fragebögen wurden am 21.04.2020 per E‑Mail versendet. Aufgrund des Charakters einer Blitzumfrage wurde das Zeitfenster für die Teilnahme bewusst auf eine Woche begrenzt. Die Ärztlichen Direktoren und Chefärzte (bzw. Lehrbeauftragten) wurden gebeten, den 8 Items umfassenden Fragebogen auszufüllen und umgehend zurückzusenden. Die Fragen fokussierten auf die aktuelle Lehrsituation in Bezug auf digitalisierte Lehrinhalte, die Unterstützung bzw. Schaffung von adäquaten Rahmenbedingungen durch die medizinische Fakultät und IT-Einrichtungen sowie auf Kenntnis analoger Situationen in anderen vergleichbaren Einrichtungen (Abb. 1). Diese Einschätzungen konnten auf einer 5‑stufigen endpunktbenannten Skala von 1 (trifft voll zu) bis 5 (trifft gar nicht zu) vermerkt werden. Ursprüngliche Intention der Berücksichtigung eines Studiendekanats und jeweils einer universitären chirurgischen und medizinischen Klinik war es, mögliche Gemeinsamkeiten und/oder Diskrepanzen in der Wahrnehmung zwischen den Institutionen respektive Fachbereichen zu berücksichtigen. Da tendenziell eine starke Übereinstimmung der Ergebnisse abzulesen war, wurden diese in der Auswertung der UK integriert. Einsendeschluss für die Fragebögen war der 29.04.2020 (Rücklauffrist 8 Tage).

Die Ergebnisse der Evaluation wurden mit SPSS (IBM SPSS Statistics für Windows, Version 24.0, Fa. IBM Corp. 2015, Armonk, NY, USA) deskriptiv ausgewertet. Die Erstellung der Tabellen erfolgte mit Excel bzw. Word (Fa. Microsoft 2010, Redmond, WA, USA).

## Ergebnisse

31 UK und 10 ALK beantworteten den Fragebogen und gingen in die Auswertung ein. Die Rücklaufquote bezogen auf die UK betrug 74,4 %, die der ALK 50 %. 3 UK sandten Fragebögen nach Ablauf der Frist ein und wurden nicht berücksichtigt. Von den Antwortenden waren 77 % Lehrbeauftragte. Die Ergebnisse der einzelnen Fragen (Mittelwerte) sind der Abb. 1 zu entnehmen. Die prozentuale Verteilung der Antwortmöglichkeiten ist in Tab. 1 sowohl für UK als auch ALK aufgeführt. Mögliche Freitextkommentare wurden thematisch kategorisiert und nach Häufigkeit gerankt.

**Table 1:** 

Frage Nr.	Zutreffend (%)	Eher zutreffend (%)	Unentschieden (%)	Eher nicht zutreffend (%)	Nicht zutreffend (%)
*1 UK*	25,8	9,7	32,3	12,9	19,4
*1 ALK*	30	20	30	0	20
*2 UK*	25,8	22,6	12,9	22,6	16,1
*2 ALK*	10	10	30	20	30
*3 UK*	29	32,3	6,5	9,7	22,6
*3 ALK*	20	20	10	40	10
*4 UK*	12,9	29	22,6	22,6	12,9
*4 ALK*	20	10	10	20	40
*5 UK*	19,4	38,7	22,6	12,9	6,5
*5 ALK*	10	20	30	20	20
*6a UK*	38,7	41,9	3,2	12,9	3,2
*6a ALK*	10	10	20	20	40
*6b UK*	16,1	35,5	16,1	19,4	12,9
*6b ALK*	11,1	0	11,1	22,2	55,6
*6c UK*	9,7	25,8	19,4	19,4	25,8
*6C ALK*	0	0	33,3	11,1	55,6
*6d UK*	3,2	3,2	19,4	32,3	41,9
*6d ALK*	0	0	0	12,5	87,5
*6e UK*	3,2	25,8	22,6	22,6	25,8
*6e ALK*	22,2	0	11,1	22,2	44,4
*7 UK*	12,9	16,1	12,9	25,8	32,3
*7 ALK*	10	0	10	50	30
*8 UK*	26,7	33,3	6,7	13,3	20
*8 ALK*	10	0	20	30	40

*UK* Universitätsklinikum, *ALK* akademisches Lehrkrankenhaus

Die prozentualen Ergebnisse ergaben in Bezug auf die Informationspolitik der MF tendenziell eher zustimmende Werte für UK und ALK, bezüglich des Zeitverlaufs aber deutlich dissoziierende Werte zum inhaltlichen Informationsgehalt (Tab. 1). 35,5 % der UK und 50 % der ALK fühlten sich mit ausreichendem zeitlichen Vorlauf vonseiten der MF über die zu erwartenden Lehrbedingungen informiert. Über das zu erwartende Szenario der reinen „Online-Lehre“ fühlten sich inhaltlich 48,4 % der UK, aber nur 20 % der ALK ausreichend informiert. Die Fragen 3–5 zur lokalen Lehrsituation zeigten sowohl Gemeinsamkeiten als auch Unterschiede zwischen UK und ALK. Die Frage 3 zur Information durch die Klinikdirektion wurde sowohl bei UK (61,3 %) als auch bei ALK (40 %) positiv beschieden. Bezüglich der Verfügbarkeit einer eigenen adäquaten digitalen Infrastruktur (Frage 4) votierten 41,9 % der UK eher für zutreffend, während demgegenüber 60 % der ALK dies eher verneinten. In der Frage 5 zu angemessenen lokalen Rahmenbedingungen, wie Akzeptanz, Unterstützung, Personal oder Investitionen, zeigten sich die ausgeprägtesten Divergenzen. Während 58,1 % der UK hier (eher) zutreffend angaben, waren 40 % auf der ALK-Seite der Meinung, dass dies (eher) nicht zutraf. In der unterteilten Frage 6 wurde die Unterstützung bei der Erstellung von Online-Lehrinhalten behandelt. Abgefragt wurde die Unterstützung durch die eigene Klinik, MF, IT der Klinik, Rechenzentren und Universitäten. Die ALK zeigten hier stringent in die negative Richtung (60–100 %), was die UK nur in Bezug auf klinikassoziierte IT, Rechenzentrum und Universität taten (45–74 %). Immerhin votierten noch 35,5 % positiv für die klinikeigene IT, während die medizinische Fakultät (51,6 %) und die eigene Klinik (80,6 %) mehrheitlich positiv beurteilt wurden. Die Frage 7 zum Vorliegen digitaler Lehrinhalte vor dem Pandemiebeginn wurden mit 58,1 % (UK) und 80 % (ALK) eindeutig als nicht zutreffend eingestuft (Abb. 1**;** Tab. 1). In der letzten Frage 8 wurde nach der Kenntnis anderer vergleichbarer Situationen im Bundesgebiet gefragt. Hierbei attestierten 60 % der UK, darüber analog Kenntnisse zu besitzen, während 70 % der ALK dies negierten (Tab. 1).

Zudem bestand die Möglichkeit, in Freitextform additive Kommentare abzugeben. Hier kristallisierten sich organisatorische, technische, inhaltliche und didaktische Aspekte heraus:

### Organisatorisch

- Unzureichende Konkretisierung des Informationsflusses vonseiten des Studiendekanats
- Unzureichende Personalressourcen in Kliniken als limitierender Faktor, aus diesem Grund erfolgte die Digitalisierung von Lehrinhalten z. T. durch (PJ-)Studierende

### Technisch

- Unzufriedenheit mit Unterstützung durch Rechenzentren aufgrund mangelnder struktureller und personeller Kapazitäten
- Uneinheitliche/widersprüchliche Argumentationen bezüglich Datenschutz bei Anwendung von Videokonferenzsystemen
- Möglichkeiten zur Live-Übertragung von Lehrveranstaltungen scheiterten an nicht ausreichender Bereitstellung von Netzkapazität

### Inhaltlich

- Aktueller „Notfallunterricht“ orientiert sich nicht am „constructive alignment“ (oder zu Deutsch „didaktische Kohärenz“) der MF. Darunter versteht man ein ausbalanciertes System, in dem die relevanten Lernaktivitäten mit den Lernzielen und deren Prüfung korrespondieren. Dies gilt unverändert in einer digitalen Lernumgebung.
- Fehlende Infrastruktur für inhaltliche Neugestaltung des Lehrangebots (Zeitspanne)
- Mangel an kurzfristig verfügbaren digitalen Lehrmaterialien

### Didaktisch

- Wunsch nach einheitlicher nationaler Gestaltung des Lehrbetriebs (ggf. unter Beteiligung der DGHNO-KHC [Deutschen Gesellschaft für Hals-Nasen-Ohren-Heilkunde, Kopf- und Hals-Chirurgie e. V.])
- Wunsch nach Fortsetzung/Aufrechterhaltung von Intensität der Digitalisierungsbestrebungen nach Ende der Corona-Pandemie
- Mangelnde kollegiale und interprofessionelle Unterstützung der didaktischen Umsetzung eines Lehrkonzepts

## Diskussion

Unsere Umfrage zeigt, dass die kurzfristige Umstellung auf eine rein digitale Lehre vielerorts mit großen Schwierigkeiten verbunden war. Es wäre eigentlich zu erwarten, dass in einer in vielen Bereichen digitalisierten Welt eine kurzfristige Umstellung auf einen virtuellen Lehrbetrieb vergleichsweise problemlos zu meistern sein sollte. Tatsächlich wird wohl unbekannt bleiben, von welchen Rahmenbedingungen die zuständigen Institutionen bei ihrer Planung ausgegangen sind. Die MF reichten diese brisante und zeitlich terminierte Aufgabe Kraft ihrer Weisungsbefugnis an die Fachbereiche/Kliniken weiter. Der Zeitpunkt dieser Delegation war bundesweit nicht einheitlich, aber doch zeitlich recht eng benachbart.

Entsprechend zeigte sich in unserer Umfrage hinsichtlich der zeitlichen Vorbereitung auf die zu erwartenden besonderen Lehrbedingungen (Rahmenbedingungen, Fristen, Prüfungen) durch die MF vor Ort ein gemischtes Bild. So gaben 35,5 % der teilnehmenden UK und 50 % der ALK an, dass sie zeitgerecht über die zu erwartenden Lehrbedingungen informiert wurden. Immerhin 32,2 % der UK und 20 % der ALK empfanden die Vorbereitungszeit jedoch als ungenügend. Über die Tatsache einer ausschließlichen Online-Lehre fühlten sich 48,4 % der UK und nur 20 % der ALK rechtzeitig informiert. Zum Teil wurde hier in den freien Kommentaren jedoch Verständnis für die wenig vorhersehbare Situation signalisiert. Aufgrund dieser Unvorhersehbarkeit befanden sich MF und ALK, die Universitäten und die dazugehörigen Supporteinrichtungen (Stand: Mai 2020) vielfach in einer reaktiven Haltung auf äußere Gegebenheiten der Corona-Krise.

Die digitale Transformation der Hochschullehre soll von allen Akteuren als ein langanhaltender disruptiver Veränderungs- und Innovationsprozess verstanden werden, der die Strukturen, Prozesse und Kulturen und damit die Rollen, Kompetenzen und Kooperationen stark verändern wird [8]. Zum jetzigen Zeitpunkt ist jedoch die Notwendigkeit zum unmittelbaren Handeln die bestimmende Kraft [3, 5], wenngleich bekannterweise ein Bedarf an digitalisierten (prä- und postgraduierten) Lehrinhalten und -konzepten, gerade in der HNO-Heilkunde, besteht [4, 21, 22]. Digitales Lernen und Lehren flächendeckend zu implementieren, setzt Prozesse auf verschiedenen Ebenen der Bildungsinstitutionen voraus: strategische Prozesse aufseiten der Leitungen, fachübergreifende Prozesse bei den Kompetenzzentren der Bildungsinstitutionen, fachliche Prozesse bei den Lehrenden [8].

Digitale Lehr- und Lernformate sind in der aktuellen Situation vielerorts die einzige Alternative, um ein Studium zu ermöglichen. Für die weitere Entwicklung des aktuellen „Notfall-Lehrbetriebs“ muss dieser Prozess aber vom didaktisch Sinnvollen und nicht nur von den äußeren Gegebenheiten bestimmt werden. Mit digitalen Lerntechnologien ist die Herausforderung für die didaktische Gestaltung gestiegen. In unserer Umfrage zeigt sich, dass der Einsatz digitaler Lehre an den beteiligten Bildungsinstitutionen sehr heterogen, jedoch insgesamt eher gering ausgeprägt war. Dies kann in der aktuellen Situation eine Reihe von Schwierigkeiten bedingen. Aufseiten der Dozierenden bestehen mitunter Defizite bei den technischen und z. T. bei den didaktischen Fertigkeiten [5, 10]. Gleichzeitig besteht für die Dozenten im ersten Schritt ein hoher Mehraufwand ohne adäquaten Support [5, 27]. Lehrende müssten für die Umsetzung digitaler Kompetenzen durch eigene Fortbildungsmaßnahmen qualifiziert und in der Umsetzung unterstützt werden. Dies umfasst sowohl neue technische Aspekte als auch digitale didaktische Methoden. „Constructive alignment“ muss auch in der digital unterstützten Lehre im Zentrum stehen.

Die Zunahme der Anforderungen durch eine Professionalisierung der Lehre macht sich auch in den Kliniken bemerkbar [2, 4, 6, 15–17, 24]. Durch die Notwendigkeiten der COVID-19-Pandemie zeigte sich, dass eine digitale Infrastruktur für höhergradige Anforderungen vielerorts quasi nicht existent ist und/oder (momentan) nicht realisiert werden kann. Diese Inkongruenz lässt sich am ehesten auf bisher nicht genutzte Möglichkeiten bei der Umsetzung der digitalen Transformation zurückführen, da der (politische) Wille zur Digitalisierung seit Jahren besteht und finanziell gefördert wird [1, 10, 11]. In unserer Umfrage gaben lediglich 29 % der Unikliniken und 10 % der ALK an, dass bereits vor der Corona-Pandemie ein umfangreiches digitales Lehrangebot vorhanden war. Bedauerlicherweise unterstreicht dies, wie wenig Progress es seit einer Umfrage an deutschen Universitäts-HNO-Kliniken 2016 zu geben scheint. Hierbei befragten von Saß et al. den Einsatz von E‑Learning ausschließlich bei den Lehrverantwortlichen und den studentischen medizinischen Fachschaften. Interessant hierbei ist, dass vonseiten der Studierenden das E‑Learning-Angebot im Gesamt-Curriculum, im Gegensatz zu den Lehrverantwortlichen, als gering bis nicht existent eingeschätzt wurde [21]. Unsere o. g. Zahlen zeigen aber auch, dass ein einige UK in puncto „digitalisierte Lehre“ schon frühzeitig Eigeninitiative entwickelt und Kooperationen gebildet haben [16]. Folglich war für diese Zentren die zeitnahe Umstellung auf Online-Lehre kein wesentliches Problem.

Die digitale Transformation scheint an deutschen UK de facto nur partiell und/oder mit Zeitverzug stattzufinden. Sie zeigt sich oftmals abhängig vom persönlichen Engagement einzelner Führungskräfte und vom berufspolitischen Willen zur interprofessionellen Kooperation (z. B. Rechenzentrum). Die digitale Infrastruktur, als Conditio sine qua non stellt sich mitunter als unerwarteterweise unvorbereitet dar und wird in den verschiedenen Kliniken in unserer Umfrage sehr unterschiedlich beurteilt. Hier zeigt sich v. a. bei den ALK ein deutlicher Bedarf. Dies ist in gewisser Weise nachvollziehbar, da die Lehre als integraler Bestandteil der Hochschulmedizin den UK zugeordnet wird. Jedoch gaben immerhin noch ein Drittel der UK an, dass eine adäquate digitale Ausstattung eher nicht vorhanden ist.

Zudem zeigte sich in unserer Umfrage, dass die Kliniken in der Vorbereitung und Erstellung der Online-Lehrinhalte meist auf sich selbst gestellt waren, mit z. T. noch etwas Unterstützung durch die MF. Scheinbar gängig war die Nutzung zusätzlicher Personalressourcen (Studierende im Praktischen Jahr, Famuli), da sie vermutlich unter den gegebenen Zeitverhältnissen am schnellsten und kostengünstigsten zu realisieren war. Vorteilhaft erscheint zudem, dass viele der Studierenden eine professionelle Lehre wertschätzen und somit weder einer besonderen Motivation noch einer technischen Einweisung bedürfen (Generation „digital natives“) [9–11]. Die geringste Unterstützung erhielten die Kliniken durch die Rechenzentren und Universitäten.

Die Corona-Krise hat Schwachpunkte aufgedeckt. Sie hat aber auch Zeitkontingente durch die Reduktion des Patientenaufkommens geschaffen, welche an mehreren Kliniken zu Mitarbeitereinteilungen für Digitalisierungs‑/Lehraufgaben geführt hat. Die Entwicklung der Corona-Rahmenbedingungen mit Erhöhung des Patientenaufkommens seit April 2020 lässt jedoch vermuten, dass dies nicht aufrechterhalten werden kann. Diese Beobachtung wird gestützt durch unsere Umfrageergebnisse: Lediglich 58,1 % der UK bzw. 30 % der ALK stimmten der Aussage zu, dass generell bezüglich der Lehre angemessene Rahmenbedingungen, wie Akzeptanz, Unterstützung, personelle Ausstattung und Investitionen, an der jeweiligen Klinik bestehen. Ohne in Extreme zu verfallen, scheint es doch zumindest ein Problem der Priorisierung zu sein, wenn es sich um Themen handelt, welche vorrangig die Lehre betreffen. Angesichts des Transformationsdrucks entstehen auch in der postgraduellen Weiterbildung digitale Curricula, die standortübergreifend Ressourcen bündeln [18].

Zur systematischen und zielgerichteten Weiterentwicklung bieten sich in Anlehnung an den Strategieprozess von Kuhn et al. [8] folgende Handlungsempfehlungen an:

### Rahmenbedingungen an Universitätskliniken und Lehrkrankenhäusern schaffen

Um eine erfolgreiche Implementierung digitaler Bildungskonzepten zu ermöglichen, sollen Bildungsinstitutionen die organisatorischen, technischen, didaktischen und personellen Rahmenbedingungen schaffen [5, 25, 26]. Von der Politik ist hierbei sicherzustellen, dass die finanziellen Mittel bereitgestellt werden und flächendeckend gleichwertiger Zugang zur digitalen Infrastruktur besteht [5, 8].

### Digitale Transformation durch Co-Design gestalten

Aktuelle und zukünftige Entwicklungen der digitalen Transformation können durch eine intensive Kooperation mit den Studierenden (Co-Design) entwickelt und implementiert werden. Sie sollten sich an den Anforderungen und Bedürfnissen der Studierenden und nicht am technisch Machbaren orientieren.

### Prozess agil entwickeln

Bei der Implementierung sollte die hohe Geschwindigkeit des derzeitigen Veränderungsprozesses berücksichtigt werden und bewusst Freiräume zur iterativen Anpassung geschaffen werden.

Es bleibt festzustellen, dass der Studiengang Humanmedizin wohl kaum im Verbund der Nicht-Präsenz-Lehre zu erwarten ist. Allein die regelhaft persönlich notwendige Vermittlung praktischer Fertigkeiten sowie die so wichtige Vermittlung von Fertigkeiten der Gesprächs- und Patientenführung sollte möglichen Plänen eines reinen „Fernstudiums“ zuwiderlaufen. Gleichwohl sind interessante „Mischformen“ der Lehre zukünftig zu erwarten.

Als Folge der bisherigen Corona-Erfahrungen und um in diesen Zeiten einen konstruktiven Beitrag zum dauerhaften Strukturaufbau zu leisten, hat sich die Arbeitsgruppe „Lehren und Prüfen in der HNO-Heilkunde“ (ArGru LuP) der Deutschen Gesellschaft für HNO-Heilkunde, Kopf- und Hals-Chirurgie (DGHNO-KHC) entschlossen, den Fundus für eine Plattform zu legen, auf welcher digitalisierte Lehrmaterialien für alle bundesweiten HNO-Kliniken kostenfrei zur Verfügung gestellt werden. Die primären Inhalte aus den UK Dresden, Frankfurt, Freiburg, Regensburg und Würzburg stehen bereits kostenfrei zur Verfügung und sollen zukünftig aufgestockt werden. Neue oder fehlende Lehrinhalte können und sollen auch von anderen Kliniken wünschenswerterweise eingestellt werden. Momentan haben bereits die UK Jena und Köln ihre Bereitschaft zur Partizipation in Aussicht gestellt. Diese zukunftsorientierte Aktion kann bereits aktuell helfen, Probleme zu lösen [5]. Sie erfolgt in Kooperation („HNO-Lehrmaterialien“) zwischen der ArGru LuP und der DGHNO-KHC.

Eine weitere erwähnenswerte Initiative kommt von der NKLM-Geschäftsstelle. Seit nunmehr 2 Jahren wird der aktuelle „Nationale kompetenzbasierte Lernzielkatalog Medizin“ (NKLM), auch unter aktiver Mitarbeit einer Task Force der DGHNO-KHC, in einem aufwendigen Prozess weiterentwickelt [14]. Dies geschieht auf einer onlinebasierten Datenbankplattform namens LOOOP (Learning Opportunities, Objectives and Outcomes Platform). Diese an der Charité entwickelte Datenbank wurde vor der notwendigen Bündelung von Ressourcen für die Sammlung und Vernetzung von virtuellen und digitalen Lehrinhalten erweitert [12]. Hier können alle MF die bereits am Standort vorhandenen digitalen Angebote auflisten und zunächst gegen den alten NKLM mappen. Die Plattform hält dabei nicht die Materialien selbst vor, sondern die Links zu den Fakultätsseiten, auf denen diese abgelegt sind. Durch direkten Absprung auf die Lernmanagementseite der Fakultäten können die Inhalte dann eingesehen werden.

## Fazit

Die Corona-Krise sollte als Chance gesehen werden, bisher Versäumtes zu verbessern und nicht genutzte Potenziale freizusetzen. Es gilt nun, adäquate Rahmenbedingungen für die professionelle, digitalisierte Lehre zu schaffen, und weniger, sich mit den aktuell implementierten Lehrinhalten einer „notfallbedingten Fernlehre“ zu arrangieren. Vielmehr sollte eine kritische Auslese der erworbenen Erfahrungen aus diesem virtuellen Semester erfolgen, um eine systematische, zielgerichtete respektive konzeptionelle Weiterentwicklung der Lehre zu gewährleisten. Die Hoffnung auf die Aufrechterhaltung des gegenwärtigen Impulses zur Digitalisierung wurde auch durch einige Teilnehmer der Umfrage formuliert. Da die Limitation dieser Studie in der fehlenden Mittelfristanalyse deutlich wird, sollte dies in einer Folgestudie erörtert werden.

## References

[1] Bundesministerium für Bildung und Forschung (2017) Digitalisierung in der Medizin. https://www.bmbf.de/de/digitalisierung-in-der-medizin-2897.html. Zugegriffen: 29. Apr. 2020

[2] Chen F, Lui AM, Martinelli SM (2017). A systematic review of the effectiveness of flipped classrooms in medical education. Med Educ.

[3] Czerniewicz L (2020) What we learnt from „Going online“ during university shutdown in South Africa. PhilOnEdTech, March 15, 2020. https://philonedtech.com/what-we-learnt-from-going-online-during-university-shutdowns-in-south-africa/. Zugegriffen: 10. Mai 2020

[4] Dombrowski T, Dazert S, Volkenstein S (2019). Digitale Strategien in der Lehre. Laryngol Rhinol Otol.

[5] Hodges C et al (2020) The difference between emergency remote teaching and Online learning. https://er.educause.edu/articles/2020/3/the-difference-between-emergency-remote-teaching-and-online-learning. Zugegriffen: 9. Mai 2020

[6] Guo PJ, Kim J, Rubin R (2014) How video production affects student engagement: an empirical study of MOOC video. http://dl.acm.org/citation.cfm?doid=2556325.2566239. Zugegriffen: 9. Apr. 2020 (ACM Press, p. 41–50)

[7] Hochschulforum Digitalisierung (2016). The Digital Turn—Hochschulbildung im digitalen Zeitalter.

[8] Kuhn S, Ammann D, Cichon I et al (2019) Careum working paper 8—long version: „Wie revolutioniert die digitale Transformation die Bildung der Berufe im Gesundheitswesen?“. www.careum.ch/workingpaper8-lang. Zugegriffen: 2. Mai 2020

[9] Kuhn S (2018). Medizin im digitalen Zeitalter: Transformation durch Bildung. Dtsch Ärztebl Int.

[10] Kuhn S, Kadioglu D, Deutsch K (2018). Data Literacy in der Medizin. Welche Kompetenzen braucht ein Arzt?. Onkologe.

[11] Kuhn S, Frankenhauser S, Tolks D (2017). Digitale Lehr- und Lernangebote in der medizinischen Ausbildung. Schon am Ziel oder noch am Anfang?. Bundesgesundheitsbl.

[12] https://looop-share.charite.de. Zugegriffen: 20. Apr. 2020

[13] Means B, Means B, Bakia M, Murphy R (2014). Online schools and universities. Learning Online: what research tells us about whether, when and how.

[14] Nationaler Kompetenzbasierter Lernzielkatalog Medizin (NKLM) für Deutschland: Zusammenarbeit der Gesellschaft für Medizinische Ausbildung (GMA) und des Medizinischen Fakultätentages (MFT): German Medical Science GMS Publishing, House; Düsseldorf; 2009

[15] Offergeld C, Neudert M, Zahnert T (2020). Einsatz und Akzeptanz des PJ-HNO-Basis-Logbuchs an deutschen Universitätskliniken und akademischen Lehrkrankenhäusern. HNO.

[16] Offergeld C, Neudert M, Emerich M (2020). Vermittlung digitaler Kompetenzen in der curricularen HNO-Lehre: abwartende Haltung oder vorauseilender Gehorsam?. HNO.

[17] Polk ML, Lailach S, Kemper M (2020). Lernkurve der HNO-Spiegeluntersuchung. HNO.

[18] Roy SF, Cecchini MJ (2020). Implementing a structured digital-based online pathology curriculum for trainees at the time of COVID-19. J Clin Pathol.

[19] Salmon G, Salmon G (2013). E-tivities in the five-stage model. E-tivities: the key to active online learning.

[20] https://www.stifterverband.org/pressemitteilungen/2020_04_20_hochschul-barometer_corona-krise. Zugegriffen: 27. Apr. 2020

[21] Freiherr von Saß P, Klenzner T, Scheckenbach K (2017). Einsatz von E-Learning an deutschen Universitäts-HNO-Kliniken. Laryngol Rhinol Otol.

[22] Shabli S, Heuermann K, Leffers D (2019). Umfrage zum Bedarf einer e-Learning-Plattform für Ärzte in der HNO-Facharzt-Weiterbildung. Laryngol Rhinol Otol.

[23] Tolks D, Schäfer C, Raupach T (2016). An introduction to the inverted/ flipped classroom model in education and advanced training in medicine and in the healthcare professions. GMS J Med Educ.

[24] Wijnen-Meijer M, Gratmeier M, Berberat PO (2020). Übersicht über die Forschung im Bereich der medizinischen Ausbildung. HNO.

[25] Wissenschaftsrat (2020) Neustrukturierung des Medizinstudiums und Änderung der Approbationsordnung für Ärzte. Empfehlungen der Expertenkommission zum Masterplan Medizinstudium 2020. https://www.wissenschaftsrat.de/download/archiv/7271-18. Zugegriffen: 18. Okt. 2019

[26] https://www.zeit.de/campus/2020-04/digitales-semester-coronavirus-studium-goethe-universitaet-frankfurt. Zugegriffen: 5. Mai 2020

[27] Zimmerman J (2020) Coronavirus and the great online-learning experiment. https://www.chronicle.com/article/Coronavirusthe-Great/248216. Zugegriffen: 9. Mai 2020 (Chronicle of Higher Education, March 10, 2020)

